# Survey of Peritoneal Dialysis Patients' Challenges and Experiences during the COVID-19 Pandemic: A Multicenter Study in the United States

**DOI:** 10.34067/KID.0000000000000202

**Published:** 2023-06-26

**Authors:** Farah AbiFaraj, Dale Lee, Meghan Lacovara, Tarun Kapoor, Rebecca Seshasai, Shweta Bansal, Robert Greevy, Andrew Guide, Shuchita Sharma, Jaime Uribarri, Osama El Shamy

**Affiliations:** 1 Division of Nephrology, Johns Hopkins University School of Medicine, Baltimore, Maryland; 2 Division of Nephrology, Icahn School of Medicine at Mount Sinai, New York, New York; 3 Renal Electrolyte and Hypertension Division, Department of Medicine, Perelman School of Medicine, University of Pennsylvania, Philadelphia, Pennsylvania; 4 Department of Nephrology and Hypertension, Vanderbilt University Medical Center, Nashville, Tennessee; 5 Division of Nephrology, Department of Medicine, University of Texas Health San Antonio, San Antonio, Texas; 6 Department of Biostatistics, Vanderbilt University Medical Center, Nashville, Tennessee

**Keywords:** chronic dialysis, dialysis, patient satisfaction, patient-centered care, peritoneal dialysis

## Abstract

**Key Points:**

- The adjustments made by the dialysis units during the peak of the pandemic were effective in maneuvering the challenges faced by our patients during the COVID-19 pandemic.
- Patients who remained on PD were satisfied with the quality of care, felt supported by the unit staff, and did not report feeling anxious or depressed.

**Background:**

During the coronavirus disease 2019 (COVID-19) pandemic, adjustments were made to peritoneal dialysis (PD) practices in the outpatient units. These were decisions made by dialysis providers, clinical staff, and governments with input from patient organizations in some instances. The patient perspective regarding these changes during the pandemic has not been explored. We sought to evaluate patient experiences and perceptions of the challenges they faced, institutional adjustments, and their mental health during the height of the COVID-19 pandemic in the United States.

**Methods:**

We conducted a cross-sectional survey of PD patients across four home dialysis units affiliated with four large academic centers, who were on PD between March 2020 and March 2021.

**Results:**

Ninety-eight participants completed the survey across the four outpatient PD clinics. Over 95% of patients did not have to change their home accommodations during the pandemic, and over 80% did not have issues getting their dialysis supplies and medications delivered. Ninety-three percent of patients felt comfortable coming to the dialysis unit if they needed to during the pandemic. Almost all patients (98%) felt supported by their dialysis staff during the peak of COVID-19 and had modified Patient Health Questionnaire 2 (91%) and Generalized Anxiety Disorder 2-item (96%) scores not consistent with depression or anxiety. Less than 10% of patients considered changing their dialysis modality.

**Conclusions:**

The adjustments made by the dialysis units during the peak of the pandemic were effective in maneuvering the challenges faced by our patients during the COVID-19 pandemic. Overall, patients who remained on PD were satisfied with the quality of care, felt supported by the unit staff, and did not report feeling anxious or depressed.

## Graphical abstract

**Figure:**
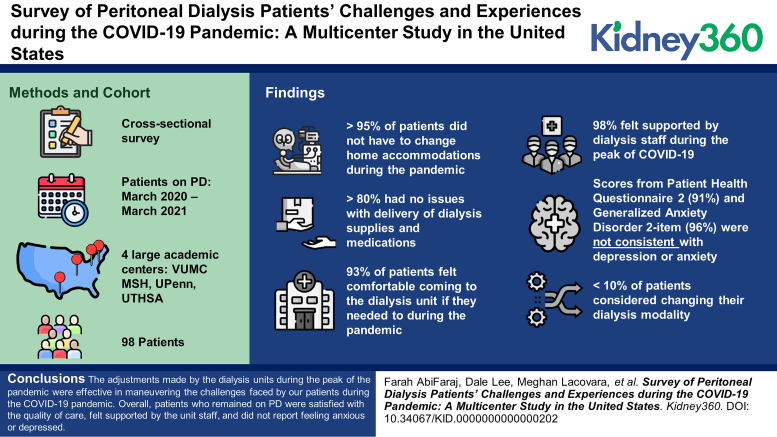

## Introduction

In 2020, severe acute respiratory syndrome coronavirus 2 caused the coronavirus disease 2019 (COVID-19) pandemic that upended the world. Hospitals were overfilled, and millions of people were affected.^1–3^ Patients with ESKD are immunocompromised^4^ and, therefore, particularly vulnerable. Social distancing and isolation were the hallmark approaches to reducing exposure during the peak of the pandemic.^5–8^ Patients on chronic peritoneal dialysis (PD) perform their dialysis treatments at home and only require monthly visits with their providers, often conducted in person in the dialysis unit. Leveraging the existing home dialysis treatment practices to minimize patients' potential exposure during treatments and adjusting clinic visit practices was a relatively easy adjusting compared with in-center dialysis patients.

Medical societies, such as the International Society of Peritoneal Dialysis, released recommendations with adjustments and best practices for providers to continue delivering both chronic and urgent-start PD during the peak of the pandemic.^9^ Many health care systems and dialysis units implemented policy changes on local, regional, and national levels to care for and protect dialysis patients, caregivers, and health care staff. These included expanding telehealth utilization and capabilities, preclinic visit screening, personal protective equipment protocols, limits on caregiver/visitor entry to the units, and changes to monthly bundle medication deliveries.^9,10^

These were decisions made by dialysis providers, physicians/clinical staff, and governments with input from ESKD patient organizations in some instances. However, the patient perspective regarding these changes during the pandemic has not been explored. The success of these measures is more than simply assessing patient mortality, COVID-19 infection rates, and modality survival. Patients play a pivotal role in their care and nowhere more than in home dialysis. If we are faced with another pandemic in the future, it is important that patient experiences and perceptions are considered.

In this multicenter survey study, we sought to evaluate patient experiences and perceptions of the challenges they faced, institutional adjustments, and patients' mental health during the height of the COVID-19 pandemic in the United States.

## Methods

### Study Design and Surveys

We conducted a cross-sectional survey across four home dialysis units affiliated with four large academic centers: Vanderbilt University Medical Center (VUMC) in Tennessee, Mount Sinai Hospital (MSH) in New York, Hospital of the University of Pennsylvania (UPenn) in Pennsylvania, and University of Texas Health, San Antonio (UTHSA), in Texas.

This study was approved by the Vanderbilt Institutional Review Board (IRB-211289) and the respective participating sites' institutional review boards. Clinical research agreements were also completed between the participating sites with VUMC as the lead site.

### Eligibility

Patients were considered eligible if they (*1*) were 18 years or older; (*2*) received PD for ≥3 months between March 1, 2020, and March 31, 2021; and (*3*) were still receiving PD in their respective units at the time of conducting the survey.

### Data Collection

Survey collection started in October 2021 and concluded in April 2022. Survey questions included baseline demographic information, questions about patients' PD experiences during the period of March 2020–March 2021, as well as modified Generalized Anxiety Disorder 2-item (GAD-2) and Patient Health Questionnaire 2 (PHQ-2) questions. The survey questions are present in Supplemental Material S1.

Study participants were approached in their home dialysis units after completing their monthly clinic visit. They were then given the paper surveys (Supplemental Material) that included no identifying questions. Survey responses were collected by the individual institutions and entered in the VUMC RedCap survey site.

### Policy Adjustments during the Pandemic

Each site had its own policies in place to help protect patients, their caretakers, and the health care staff. All four facilities had a prescreening policy, required masks before entering the unit, and did not allow family members in the waiting room. All patients with issues outside of the monthly clinic visits were triaged through a phone call with the nursing staff/nephrologist and instructed to come in if needed. Things that differentiated some units from the rest included that VUMC had designated blocked seats in the waiting room and subsequently did not perform scheduling changes to avoid full waiting rooms. UTHSA patients' medications were picked up by patients from the dialysis unit. MSH contracted with a courier service that picked up patients' medications from the dialysis unit and delivered it to their homes. A contracted laboratory company obtained, delivered, and processed patients' monthly bloodwork from the comfort of their homes. A comprehensive summary of the policies of the individual participating centers are presented in Table 1.

**Table 1.** Site accommodations and adjustments during between March 2020 and March 2021

Medical Institution	Family in the Waiting Room	Blocked Seats in the Waiting Room	Prescreening Policy	Scheduling Changes to Avoid Full Waiting	Family Members Allowed Inside	PPE	Issues Outside of Monthly Visit	Bundle Medications	Telehealth	Laboratory Test Results Draws
VUMC	No	Yes	Yes	No	No	Masks	Phone calls, come in if needed	Delivery (no change)	No	In clinic
UTSA	No	No	Yes	Yes	No	Masks	Phone calls, come in if needed	Pick-up in clinic (no change)	Offered, but very limited use	In clinic
MSH	No	No	Yes	Yes	No	Masks	Phone calls, come in if needed	Delivery (pick-up in clinic before the pandemic)	Yes	Home
UPenn	No	No	Yes	Yes	No	Masks	Phone calls, come in if needed	Delivery (pick-up in clinic before the pandemic)	Yes	In clinic

PPE, personal protective equipment; VUMC, Vanderbilt University Medical Center; UTSA, University of Texas Health, San Antonio; MSH, Mount Sinai Hospital; UPenn, Hospital of the University of Pennsylvania.

### Statistical Analyses

Chi-square tests for independence were used to determine whether the proportional breakdown of the row variables was similar across all column variables (*e.g.*, for this study, one test conducted was determining whether the proportion of those who used telehealth differed across any one of the three institutions). The same chi-square tests were used to cross-tabulate the effect of different variables on telehealth use. All *P*-values were calculated using a Monte Carlo simulation to account for the small sample sizes. The Monte Carlo simulation follows the framework of the Fisher exact test, where contingency tables that preserve the table's margins are randomly generated.^11,12^ *P*-values < 0.05 were considered statistically significant. All analyses were conducted in R 4.2.0. Of note, UTSA participant results were excluded from the intersite analysis because of the relatively low number of participants.

## Results

### Demographics and Background

A total of 98 participants completed the survey across the four outpatient PD clinics (38 participants from VUMC, 32 from Mount Sinai, 19 from UPenn, and 9 from UTHSA). Patients who completed the survey accounted for over 95% of current PD patients who qualified for the study in the participating clinics. Baseline demographic and background information are summarized in Table 2. Most of the participants were male (57%), Black (43%), and older than 45 years (81%). Only 24% were working for pay during the March 2020–March 2021 period, with most of those working on-site. Most patients did not live alone (75%), but only 28% of them lived with an essential worker.

**Table 2.** Intersite comparison: Baseline characteristics and demographics of study participants

Demographic Information	MSH	UPenn	VUMC	UTHSA	Overall	*P* Value^a^
	*N*=32	*N*=19	*N*=38	*N*=9	98	
**Sex**						0.324
Male	53.1% (17)	73.7% (14)	50% (19)	67% (6)	57.1% (56)	
Female	46.9% (15)	26.3% (5)	44.7% (17)	33% (3)	40.8% (40)	
Missing	0% (0)	0% (0)	5.3% (2)	0% (0)	2.0% (2)	
**Age**						^b^
18–24	0% (0)	0% (0)	2.6% (1)	0% (0)	1.0% (1)	
25–34	3.1% (1)	5.3% (1)	7.9% (3)	11% (1)	6.1% (6)	
35–44	9.4% (3)	21.1% (4)	0% (0)	22% (2)	9.2% (9)	
45–54	25% (8)	31.6% (6)	13.2% (5)	22% (2)	21.4% (21)	
55–64	28.1% (9)	15.8% (3)	26.3% (10)	22% (2)	24.5% (24)	
65 or older	31.2% (10)	26.3% (5)	44.7% (17)	22% (2)	34.7% (34)	
Missing	3.1 (1%)	0% (0)	5.3% (2)	0% (0)	3.1% (3)	
**Ethnicity**						^b^
Non-Hispanic White	9.4% (3)	31.6% (6)	44.7% (17)	22% (2)	28.6% (28)	
Hispanic White	3.1% (1)	0% (0)	5.3% (2)	33% (3)	6.1% (6)	
Black	53.1% (17)	63.2% (12)	34.2% (13)	0% (0)	42.9% (42)	
Hispanic	28.1% (9)	0% (0)	5.3% (2)	0% (0)	11.2% (11)	
Asian	3.1% (1)	5.3% (1)	2.6% (1)	0% (0)	3.1% (3)	
Pacific islander	0% (0)	0% (0)	2.6% (1)	0% (0)	1.0% (1)	
Other	0% (0)	0% (0)	5.3% (2)	44% (4)	6.1% (6)	
None specified	3.1% (1)	0% (0)	0% (0)	0% (0)	1.0% (1)	
**Work or school during the pandemic**						0.533
Retired	34.4% (11)	21.1% (4)	34.2% (13)	0% (0)	28.6% (28)	
Disabled	28.1% (9)	26.3% (5)	28.9% (11)	22% (2)	23.4% (23)	
Working for pay	15.6% (5)	26.3% (5)	28.9% (11)	22% (2)	23.4% (23)	
Staying at home/homemaker	6.2% (2)	5.3 (1%)	10.5% (4)	44% (4)	11.2% (11)	
Laid off or lost job	6.2% (2)	0% (0)	2.6% (1)	0% (0)	3.1% (3)	
On leave	0% (0)	5.3% (1)	2.6% (1)	0% (0)	2.0% (2)	
Unemployed and looking for a job	6.2% (2)	0% (0)	0% (0)	11% (1)	3.1% (3)	
Enrolled in school, college, or university	3.1% (1)	0% (0)	0% (0)	0% (0)	1.0% (1)	
**Area of residence**						<0.001
Large city	75% (24)	63.2% (12)	15.8% (6)	67% (6)	49.0% (48)	
Suburbs	6.2% (2)	21.1% (4)	42.1% (16)	22% (2)	24.4% (24)	
Rural area	3.1% (1)	10.5% (2)	23.7% (9)	11% (1)	13.2% (13)	
Small city	9.4% (3)	5.3% (1)	13.2% (5)	0% (0)	9.2% (9)	
Town or village	6.2% (2)	0% (0)	5.3% (2)	0% (0)	4.1% (4)	
**Education**						^b^
Graduate or professional degree	9.4% (3)	5.3% (1)	21.1% (8)	0% (0)	12.2% (12)	
Some school beyond college	3.1% (1)	0% (0)	5.3% (2)	0% (0)	3.1% (3)	
4-yr college graduate	9.4% (3)	21.1% (4)	13.2% (5)	0% (0)	12.2% (12)	
Some college or 2-yr degree	25% (8)	31.6% (6)	23.7% (9)	33% (3)	26.5% (26)	
High school diploma or GED	34.4% (11)	26.3% (5)	34.2% (13)	44% (4)	33.7% (33)	
Some high school	12.5% (4)	15.8% (3)	0% (0)	22% (2)	9.2% (9)	
Some grade school	6.2% (2)	0% (0)	2.6% (1)	0% (0)	3.1% (3)	
**Income**						^b^
≥$100,000	3.1% (1)	15.8% (3)	21.1% (8)	0% (0)	12.2% (12)	
$50,000–$99,999	15.6% (5)	26.3% (5)	31.6% (12)	33% (3)	25.5% (25)	
$25,000–$49,999	28.1% (9)	31.6% (6)	31.6% (12)	44% (4)	31.6% (31)	
≤$25,000	43.8% (14)	15.8% (3)	15.8% (6)	22% (2)	25.5% (25)	
Missing	9.4% (3)	10.5% (2)	0% (0)	0% (0)	5.1% (5)	
**Essential workers living at home**						0.04
Yes	12.5% (4)	31.6% (6)	39.5% (15)	22% (2)	27.6% (27)	
No	87.5% (28)	68.4% (13)	57.9% (22)	78% (7)	71.4% (70)	
Missing	0% (0)	0% (0)	2.6% (1)	0% (0)	1.0% (1)	
**Travel time to a dialysis unit** (min)						0.112
Minimum	2	5	2	0	2	
Q1	30	20	20	8	20	
Median	45	25	40	23	30	
Q3	60	30	75	34	60	
Maximum	120	105	270	45	270	
Mean	49.7	33	61.7	21.7	50.7	
SD	32.4	26.9	63	14	47.8	
Missing	6	0	5	3	14	
**Lives alone**						0.062
Yes	40.6% (13)	26.3% (5)	15.8% (6)	11% (1)	25.5% (25)	
No	59.4% (19)	73.7% (14)	84.2% (32)	89% (8)	74.4% (73)	
**Someone to help with health needs**						0.341
Yes	65.6% (21)	73.7% (14)	81.6% (31)	78% (7)	74.5% (73)	
No	34.4% (11)	26.3% (5)	18.4% (7)	22% (2)	25.5% (25)	

MSH, Mount Sinai Hospital; UPenn, Hospital of the University of Pennsylvania; VUMC, Vanderbilt University Medical Center; UTHSA, University of Texas Health, San Antonio; GED, general educational development.

a *P*-values were calculated using Monte Carlo simulation to account for the relatively small participant sample size. UTSA participant results were excluded from this analysis because of the relatively low number of participants.

b Grouped intersite comparisons for these variables are reported in Table 3.

Approximately half of the participants reported living in a large city, with most of the rest living in the suburbs of a large city or a rural area. Over half of the patients attended some college or completed a 2-year degree (55%). The median income was $25,000–49,999/yr.

### Supplies and Accommodations

Most of the patients did not have issues getting their dialysis supplies delivered to their homes (to their doorstep, front porch, or outside their building) or into their homes (86% and 77%, respectively). Most of the patients had delivery drivers bring their supplies into their homes (69%), with family members being the second most common at 17%. Over 80% of participants had no problems getting their dialysis medications (part of the ESRD Prospective Payment System bundle) during the study period. Moreover, 95% did not need to arrange for different home accommodations to do their home dialysis treatments.

### Hesitancy to Come to the Dialysis Unit during the COVID-19 Surge

Most of the patients (87%) were not afraid to come to the dialysis unit. In fact, 93% of participants were rarely or not at all hesitant to come to the dialysis unit for their monthly visits during the height of the pandemic. When comparing institutions, participants from MSH were the only ones who showed some hesitancy in coming to the dialysis unit (19% of their cohort). These observations were replicated when assessing participants' fear of coming to the unit for dialysis-related issues due to COVID-19. Mount Sinai had the largest proportion of participants who did report having some fear (25% of their participants).

When assessing patient's modified PHQ-2 and GAD-2 scores, most of the patients had a score of ≤3 (91% and 96%, respectively), not meeting criteria for the likelihood of depression or anxiety.

### Dialysis Modality Change, Transportation, Compliance, and Transplant

Only 9% of participants considered switching to in-center hemodialysis (HD). If offered a kidney transplant during the study period, 83% of participants reported that they would have accepted. Over 94% of patients reported missing no more than 1–2 treatments per month during the period of interest.

### Intersite Analysis

Owing to the low number of patients enrolled at UTHSA (*n*=9), intersite analysis was conducted among the remaining three participating sites (*n*=89). There was no statistically significant difference between the sites in sex, age, employment, education level, work location, issues getting supplies and medications to patients' homes, fear of coming to the dialysis unit for dialysis-related issues that arose outside of their monthly visits, missed PD treatments, support from dialysis staff, consideration of switching to HD, or willingness to accept a kidney transplant (Table 2 and Supplemental Table S1). There was a higher proportion of Black and Hispanic patients in the study cohort compared with other ethnicities with statistically significant variability between the participating sites (Table 3).

**Table 3.** Group intersite comparison of select demographic, background, and day-to-day management of peritoneal dialysis^a^

Patient Characteristics and Responses	MSH	UPenn	VUMC	Overall	*P* Value^b^
**Age**					0.260
<65	65.6% (21)	73.7% (14)	50% (19)	60.7% (54)	
65 or older	31.2% (10)	26.3% (5)	44.7% (17)	36% (32)	
Missing	3.1% (1)	0% (0)	5.3% (2)	3.4% (3)	
**Ethnicity**					0.002
Black and/or Hispanic	84.4% (27)	63.2% (12)	44.7% (17)	62.9% (56)	
White, Asian, and others	15.6% (5)	36.8% (7)	55.3% (21)	37.1% (33)	
**Education**					0.410
Greater than high school	46.9% (15)	57.9% (11)	63.2% (24)	56.2% (50)	
High school or below	53.1% (17)	42.1% (8)	36.8% (14)	43.8% (39)	
**Income**					0.013
>$50,000	18.8% (6)	42.1% (8)	52.6% (20)	38.2% (34)	
<$50,000	81.2% (26)	57.9% (11)	47.4% (18)	61.8% (55)	
**Issues getting supplies delivered to home**					0.576
All the time or often	3.1% (1)	0% (0)	0% (0)	1.1% (1)	
Rarely or not at all	96.9% (31)	100% (19)	100% (38)	98.9% (88)	
**Issues getting supplies delivered inside home**					0.907
All the time or often	9.4% (3)	5.3% (1)	10.5% (4)	9% (8)	
Rarely or not at all	90.6% (29)	94.7% (18)	89.5% (34)	91% (81)	
**Trouble with getting medications**					0.253
0–4 times	100% (32)	89.5% (17)	97.4% (37)	96.6% (86)	
5 or more times	0% (0)	10.5% (2)	2.6% (1)	3.4% (3)	
**Hesitant to come to the dialysis unit**					0.004
Always or often	18.8% (6)	0% (0)	0% (0)	6.7% (6)	
Rarely or not at all	81.2% (26)	100% (19)	100% (38)	93.3% (83)	
**Fear of coming to the unit because of COVID-19**					0.511
Yes	21.9% (7)	5.3% (1)	7.9% (3)	12.4% (11)	
No	78.1% (25)	94.7% (18)	89.5% (34)	87.5% (77)	
Missing	0% (0)	0% (0)	2.6% (1)	1.1% (1)	
**Support from home dialysis staff**					0.358
All the time or often	100% (32)	100% (19)	94.7% (36)	97.8% (87)	
Rarely	0% (0)	0% (0)	5.3% (2)	2.2% (2)	
**Consider switching to in-center HD**					0.794
All the time or often	3.1% (1)	0% (0)	5.3% (2)	3.4% (3)	
Rarely or not at all	96.9% (31)	100% (19)	94.7% (36)	96.6% (86)	
**Number of missed treatments (per mo)**					1.000
<3	93.8% (30)	94.7% (18)	94.7% (36)	94.4% (84)	
3 or more	6.2% (2)	5.3% (1)	5.3% (2)	5.6% (5)	
**PHQ-2 score**					1.000
3+	12.5% (4)	5.3% (1)	7.9% (3)	9% (8)	
<3	87.5% (28)	94.7% (18)	92.1% (35)	91% (81)	
**GAD-2 score**					
3+	0% (0)	10.5% (2)	5.3% (2)	4.5% (4)	
<3	100% (32)	89.5% (17)	94.7% (36)	95.5% (85)	

MSH, Mount Sinai Hospital; UPenn, Hospital of the University of Pennsylvania; VUMC, Vanderbilt University Medical Center; COVID, coronavirus disease; HD, hemodialysis; PHQ-2, Patient Health Questionnaire 2; GAD-2, Generalized Anxiety Disorder 2-item.

a UTSA participant results were excluded from this analysis because of the relatively low number of participants.

b *P*-values were calculated using Monte Carlo simulation to account for the relatively small participant sample size.

The number of patients at MSH whose income was <$50,000 was statistically significantly higher than both at VUMC and UPenn (81.2% versus 47.4% and 57.9%, respectively, *P* = 0.01). A higher proportion of MSH patients did not live with an essential worker when compared with VUMC and UPenn (87.5% versus 68.4% and 57.9%, respectively, *P* = 0.04). VUMC patients had a lower percentage of patients who lived in large cities compared with MSH and UPenn (15.8% versus 75.0% and 63.2%, *P* < 0.001). A higher proportion of MSH patients were hesitant to come to the dialysis unit for their monthly visits when compared with VUMC and UPenn (18.8% versus 0.0% and 0.0%, *P* = 0.004).

### Travel and Transportation

Most patients (88%) did not have transportation issues coming to their dialysis unit. The mean travel time to the unit was 51 minutes (mins), with VUMC having patients with the longest travel times compared with MSH and UPenn (61.7 versus 33.0 and 49.7 mins, respectively).

### Telehealth

Sixty-eight percent of UPenn participants and 75% of MSH participants reported using telehealth all the time or often. By contrast, at VUMC, only 11% of patients reported using telehealth. Overall, 46% of participants reported using telehealth all the time or often. Telehealth analysis was conducted using data from MSH and UPenn (*n*=51), the two sites who implemented telehealth during the pandemic for their monthly clinic visits (Supplemental Table S2). Analysis of patients younger than 65 years versus older, high school education or less versus college onwards, and income <$50,000 versus ≥$50,000 showed no statistically significant difference between the groups.

### Support from Dialysis Staff and Mental Health Scoring

Almost all participants (98%) felt supported by the dialysis staff often or all the time. This did not differ between institutions.

## Discussion

In our survey of 98 participants across four large academic outpatient PD clinics, we describe the variety of changes implemented at the height of the COVID-19 pandemic to protect patients and staff. With these changes, we found that participants did not experience issues having supplies delivered to their homes, receiving medicines, or missing treatments. If they needed to come to the unit, most felt comfortable doing so and did not have issues with transportation.

There has been a growing emphasis on patient-reported outcomes when studying clinical interventions or, if possible, before intervention implementation. With the push for increasing home dialysis utilization in the United States—at the government, legislative, and provider levels—it is important to ensure that we are delivering care that aligns with patient needs. Several studies were published about recommendations for adjustments during this period^10,13–15^; however, most providers did not have time to wait for these publications and dialysis centers worldwide were making their changes simultaneously. To the best of our knowledge, there has been no publication exploring patient-reported experiences with these interventions. In the event that we are faced with another pandemic, it is important that patient experiences and perceptions are taken into consideration.

**Figure 1.**
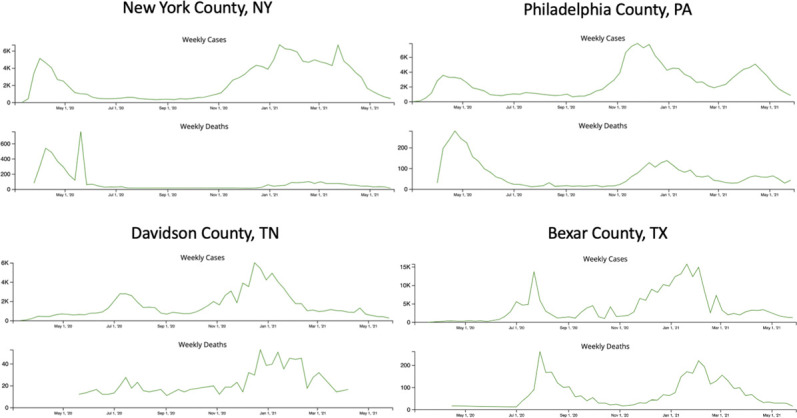
COVID-19 infection rates between March 2020 and March 2021 in different counties of the participating hospitals.

We conducted this survey in four outpatient home dialysis units in four different academic institutions across Nashville, New York City, Philadelphia, and San Antonio. The period of March 2020–March 2021 was chosen because it encompassed the worst periods of the COVID-19 pandemic in the United States. Each of the aforementioned cities had different rates of COVID-19 infection and hospitalization during the pandemic^16^ and thus different experiences (Figure 1).

In the midst of all of this, PD patients had the advantage of being able to perform their dialysis at home while using telehealth for their monthly visits.^17^ In fact, PD patients in the United States had lower rates of hospitalization because of COVID-19 than in-center HD patients.^18^ A study across four large medical institutions in Wuhan, China, showed no difference in COVID-19 infection rates between PD patients and the general population.^19^

Patients who completed the survey accounted for over 95% of current PD patients who qualified for the study in the participating clinics. The study population was representative of the dialysis populations in the cities in which the units were located. Just over half the patients were male, and the age range of the majority of our patient representative of the overall dialysis population in the United States. The study population was also very diverse, with over 70% of patients being Black, Hispanic, Asian, or Pacific Islander. The mean employment rates in dialysis patients was 23%–26%^20,21^; this was in line with our patient population where 24% were working for pay between March 2020 and March 2021. The income of our patient population was also in line with the median income in the United States.^22^ Patients traveled a mean of 51 minutes to get to their dialysis unit, far exceeding the previously reported mean time of 7.5 minutes.^23^

New York City had the highest proportion of infections and hospitalizations in the United States during the first wave of the pandemic.^24^ It is, therefore, not surprising that patients at MSH showed greater hesitance in coming to the dialysis unit because of fear of COVID-19 than participants from the other institutions.

Most of the participants (92.9%) reported missing no more than two treatments per month on average. Similarly, Gill *et al.* found increased adherence to in-center HD during the period of March–May 2020 and postulated that it was because of a desire to not be hospitalized.^25^ Adherence before and after the pandemic in our participants is beyond the scope of this study, but it is clear that participants were generally adherent to their treatments during the height of the pandemic.

Before the COVID-19 pandemic, there were measures taken to encourage the use of telehealth for home dialysis patients in the United States. Prevalent PD patients who have been on the modality for 3 months or more could conduct two of every three monthly visits through telehealth.^17^ Reported patient satisfaction with telehealth in this setting has been favorable.^26,27^ However, utilization of telehealth was limited.^25,26^ The COVID-19 pandemic accelerated interest and utilization of telehealth to deliver care all over the world.^27–31^ In our study, 41.8% of participants used telehealth often or all the time during the period of interest. Two institutions (UPenn and MSH) required telehealth visits during the first months of the pandemic.

There are many perceived barriers to telehealth usage. Among them are access to internet, limited ability to use or navigate the technology, concerns for privacy, and concern that patients will not get as good care.^32^ Older patients and their ability to use telehealth services were of particular concern, and many elderly individuals cite difficulty of use as a barrier to technology for them.^33^ One-third of individuals who stated they would never consider telehealth were older than 55 years.^32^ A prior study showed no difference in long-term satisfaction between patients who used only telemedicine and those with traditional visits, even after controlling for age and education level.^34^ This is consistent with our findings in which there was no difference in telehealth use between ages, education, income, or ethnicity. We acknowledge that this finding was skewed by the treating institution's telehealth implementation policies at that time. The lack of regulation regarding the usage of a Health Insurance Portability and Accountability Act-compliant platform during the peak of the pandemic also plays a role in the accessibility and generalizability of these results.

**Figure 2.**
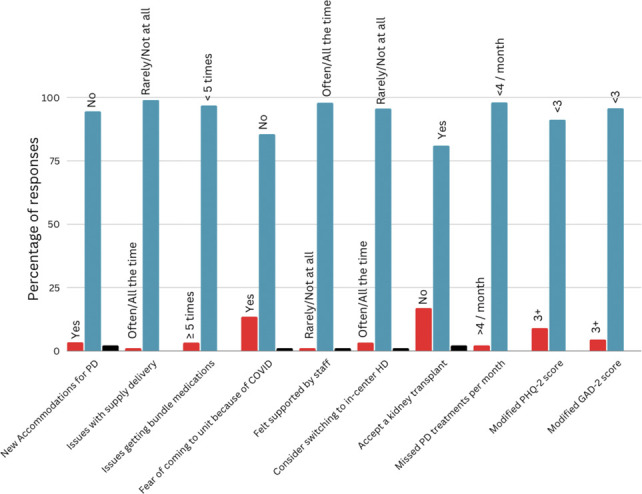
**Summary of the most important findings from the intersite analysis.** COVID, coronavirus disease; GAD-2, Generalized Anxiety Disorder 2-item; HD, hemodialysis; PD, peritoneal dialysis; PHQ-2, Patient Health Questionnaire 2.

Patients on dialysis experience a heavy burden of financial, social, and health-related pressures. Increased incidence and prevalence of depression in patients with ESKD compared with the general population has been established.^35–37^ A large study of patients with ESKD found that more than one-third of patients experienced depressive symptoms regardless of modality, except in the United States where the prevalence was 28%.^35^ The pandemic added additional stressors, such as fear of infection, fear of missing treatments and its consequences, as well as fear of exposing their loved ones. In a large study of in-center HD patients, 27% of participants reported depressive symptoms.^36^ This was consistent with reported Dialysis Outcomes and Practice Patterns Study/Peritoneal Dialysis Outcomes and Practice Patterns Study rates. Some of these factors were mitigated by adjustments in the policies and procedures during the pandemic. We found that most of the participants (>90%) across all four centers did not endorse feelings of depression or anxiety during the height of the pandemic. While this was not assessed in our study, a survey of 85 PD patients in the United Kingdom yielded 31 responses, where 58% of respondents felt negatively/ambivalent about the future.^38^ A summary of our most important findings is presented in Figure 2.

### Strengths and Limitations

Our study has several strengths. To the authors' knowledge, this is the first study surveying PD patients on practical aspects of their dialysis care, mental health, and support by their respective providers. It is a multicenter study with sites from three different regions of the United States (Northeast, Central, and South) with different rates and trends in COVID-19 case incidence, prevalence, and severity. This allowed us to capture different patient perspectives representative of different regions in the country. The study population was representative of the dialysis populations in the cities in which the units were located.

Our study has several limitations. It is a retrospective survey study encompassing the period of March 2020–March 2021, but it was conducted during October 2021–April 2022; therefore, it is subject to recall and reporting bias from the participants. There is also selection bias because patients who were on PD during the study period but were no longer on PD during survey administration were not included in the study. We did not ask patients whether they were infected with COVID-19 during the study; however, infection rates among the patients with ESKD across the different modalities have already been reported extensively. The four centers participating in this study are large home dialysis units with capabilities and resources that may not necessarily be available to smaller units around the country.

In conclusion, we found that most PD patients enrolled in the units surveyed did not have to change their home accommodations during the pandemic or have issues getting their dialysis supplies and medications delivered. Most of the patients felt comfortable coming to the dialysis unit if they needed to during the pandemic. Most of the patients felt supported by their dialysis staff during the peak of COVID-19 and had modified PHQ-2 and GAD-2 scores not consistent with depression or anxiety. Finally, <10% of patients considered changing their dialysis modality. Our survey findings demonstrate that the adjustments made by the dialysis units during the peak of the pandemic were effective in maneuvering the challenges faced by our patients during the COVID-19 pandemic while providing high-quality medical care.

## Disclosures

S. Bansal reports the following: Consultancy: Baxter; Research Funding: 3ive labs; AstraZeneca; Bayer; BI, NovoNordisk; Honoraria: UpToDate; Home Dialysis University; Advisory or Leadership Role: Section editor: *Clinical Nephrology Journal*; Editorial board member: *Kidney360*, *CJASN*; and Speakers Bureau: Home Dialysis University; Bayer Speaker Program. O. El Shamy reports the following: Employer: Vanderbilt University Medical Center; Consultancy: Outset Medical; Honoraria: Home Dialysis University; UpToDate; and Other Interests or Relationships: Home Dialysis Academy of Excellence. R. Seshasai reports the following: Employer: Atria; Clearco, Compass; Consultancy: Atria; Ownership Interest: Clearco; Compass; Sanderling; and Advisory or Leadership Role: Volition. All remaining authors have nothing to disclose.

## Supplementary Material

## Data Availability

All data is included in the manuscript and/or supporting information.

## Supplemental Material

Supplemental Table 1. Telehealth usage adjusting for demographic and background factors.

Supplemental Table 2. Intersite comparison: Changes in peritoneal dialysis-related day-to-day management between March 2020 and March 2021.

Peritoneal Dialysis Patient Survey.
