# Supplementary material for: Survey of Peritoneal Dialysis Patients' Challenges and Experiences during the COVID-19 Pandemic: A Multicenter Study in the United States
Source: Kidney360. 2023 Jun 26;4(9):e1276–85. doi: 10.34067/KID.0000000000000202 (PMC10547227; doi:10.34067/KID.0000000000000202)
Supplement: Supplementary file 1 [file kidney360-4-e1276-s001.pdf]

Table of Contents

- Supplemental Table 1. Telehealth usage in different demographic and background factors
- Supplemental Table 2. Inter-site comparison: Changes in peritoneal dialysis-related day-to-day management between March 2020 and March 2021
- Peritoneal Dialysis Patient Survey

Supplemental Table 1. Telehealth usage in different demographic and background factors*

**Variable**	**Frequently**	**Rarely or Never**	p-value**
**Age**			
<65	27	8	0.475
65+	10	5	
**Education level**			
Greater than high school	18	8	0.739
High school or below	19	6	
**Income**			
>$50,000	10	4	1.000
<$50,000	27	10	
**Race/Ethnicity**			
Black & Hispanic	21	8	1.000
White, Asian, and Others	16	6	

*Only participants from Mount Sinai Hospital and Hospital of the University of Pennsylvania were included in this analysis.
**p-values were calculated using Monte Carlo simulation to account for the relatively small participant sample size.

Supplemental Table 2. Inter-site comparison: Changes in peritoneal dialysis-related day-to-day management between March 2020 and March 2021*

	Mount Sinai Hospital	University of Pennsylvania	Vanderbilt University Medical Center	Overall	p-value**
	N = 32	N = 19	N = 38	89	
**New accommodations for Home Dialysis**					0.414
Yes	0% (0)	5.3% (1)	5.3% (2)	3.4% (3)	
No	100% (32)	84.2% (16)	94.7% (36)	94.4% (84)	
Missing	0% (0)	10.5% (2)	0% (0)	2.2% (2)	
**Work Location**					0.239
On-site	9.4% (3)	15.8% (3)	18.4% (7)	14.6% (13)	
At home	3.1% (1)	10.5% (2)	23.7% (9)	13.5% (12)	
Combination of on-site and home	9.4% (3)	5.3% (1)	2.6% (1)	5.6% (5)	
None (not working)	75% (24)	68.4% (13)	52.6% (20)	64% (57)	
Other	3.1% (1)	0% (0)	2.6% (1)	2.2% (2)	
**Telehealth for Monthly Visits**					***
All the time	28.1% (9)	21.1% (4)	7.9% (3)	18% (16)	
Often	46.9% (15)	47.4% (9)	2.6% (1)	28.1% (25)	
Rarely	15.6% (5)	26.3% (5)	34.2% (13)	25.8% (23)	
Not at all	6.2% (2)	5.3% (1)	55.3% (21)	27% (24)	
Missing	3.1% (1)	0% (0)	0% (0)	1.1% (1)	
**Issues Getting Supplies Delivered to Home**					0.576
All the time	0% (0)	0% (0)	0% (0)	0% (0)	
Often	3.1% (1)	0% (0)	0% (0)	1.1% (1)	
Rarely	9.4% (3)	10.5% (2)	13.2% (5)	11.2% (10)	
Not at all	87.5% (28)	89.5% (17)	86.8% (33)	87.6% (78)	
**Issues Getting Supplies Delivered Inside Home**					***
All the time	3.1% (1)	5.3% (1)	2.6% (1)	3.4% (3)	
Often	6.2% (2)	0% (0)	7.9% (3)	5.6% (5)	

Rarely	9.4% (3)	21.1% (4)	7.9% (3)	11.2% (10)	
Not at all	81.2% (26)	73.7% (14)	78.9% (30)	78.7% (70)	
Missing	0% (0)	0% (0)	2.6% (1)	1.1% (1)	
**Personnel Delivering Supplies Into the Home**					
Delivery Driver	84.4% (27)	73.7% (14)	50% (19)	67.4% (60)	
Family Member	9.4% (3)	15.8% (3)	26.3% (10)	18% (16)	
Friend	3.1% (1)	0% (0)	0% (0)	1.1% (1)	
Myself	0% (0)	10.5% (2)	13.2% (5)	7.9% (7)	
Other	0% (0)	0% (0)	5.3% (2)	2.2% (2)	
Missing	3.1% (1)	0% (0)	5.3% (2)	3.4% (3)	
**Trouble with Getting Medications**					***
More than 7 times	0% (0)	5.3% (1)	0% (0)	1.1% (1)	
5-7 times	0% (0)	5.3% (1)	2.6% (1)	2.2% (2)	
1-4 times	9.4% (3)	21.1% (4)	7.9% (3)	11.2% (10)	
Zero	90.6% (29)	68.4% (13)	89.5% (34)	85.4% (76)	
**Hesitant to Come to Dialysis Unit for Monthly Visits**					***
Always	0% (0)	5.3% (1)	0% (0)	1.1% (1)	
Often	18.8% (6)	0% (0)	0% (0)	6.7% (6)	
Rarely	12.5% (4)	0% (0)	18.4% (7)	12.4% (11)	
Not at all	68.8% (22)	94.7% (18)	81.6% (31)	79.8% (71)	
**Fear of Coming to Dialysis Unit because of COVID**					0.056
Yes	25% (8)	5.3% (1)	7.9% (3)	13.5% (12)	
No	75% (24)	94.7% (18)	89.5% (34)	85.4% (76)	
Missing	0% (0)	0% (0)	2.6% (1)	1.1% (1)	
**Support from Home Dialysis Staff**					***
All the time	90.6% (29)	100% (19)	92.1% (35)	93.3% (83)	
Often	9.4% (3)	0% (0)	2.6% (1)	4.5% (4)	
Rarely	0% (0)	0% (0)	2.6% (1)	1.1% (1)	
Missing	0% (0)	0% (0)	2.6% (1)	1.1% (1)	

**Consider Switching to In-Center HD**					***
All the time	3.1% (1)	0% (0)	2.6% (1)	2.2% (2)	
Often	0% (0)	0% (0)	2.6% (1)	1.1% (1)	
Rarely	6.2% (2)	0% (0)	5.3% (2)	4.5% (4)	
Not at all	90.6% (29)	100% (19)	86.8% (33)	91% (81)	
Missing	0% (0)	0% (0)	2.6% (1)	1.1% (1)	
**Accept Transplant**					0.156
Yes	84.4% (27)	89.5% (17)	73.7% (28)	80.9% (72)	
No	9.4% (3)	10.5% (2)	26.3% (10)	16.9% (15)	
Missing	6.2% (2)	0% (0)	0% (0)	2.2% (2)	
**Number of Treatments Missed** (per month)					
>4	3.1% (1)	0% (0)	2.6% (1)	2.2% (2)	
3-4	3.1% (1)	5.3% (1)	2.6% (1)	3.4% (3)	
1-2	37.5% (12)	21.1% (4)	13.2% (5)	23.6% (21)	
None	56.2% (18)	73.7% (14)	81.6% (31)	70.8% (63)	
**Transportation Issues to Dialysis Unit**					0.763
Yes	12.5% (4)	15.8% (3)	7.9% (3)	11.2% (10)	
No	87.5% (28)	84.2% (16)	92.1% (35)	88.8% (79)	

*UTSA participant results were excluded from this analysis due to the relatively low number of participants.
**p-values were calculated using Monte Carlo simulation to account for the relatively small participant sample size.
***Grouped inter-site comparisons for these variables are reported in Table 4.

Peritoneal Dialysis Patient Survey

Background:

1. **What gender do you identify with?**

☐ Man	☐ Woman	☐ Other

2. **What is your age?**

☐ 18-24	☐ 25-34	☐ 35-44	☐ 45-54	☐ 55-64	☐ 65 or older

3. **What is your race? (Please select all that apply)**
   ☐ Black or African American
   ☐ White
   ☐ American Indian or Alaska Native
   ☐ Asian
   ☐ Native Hawaiian or Other Pacific Islander
   ☐ Other: _________________

4. **Are you of Hispanic or Latino descent - that is, Mexican, Mexican American, Chicano, Puerto Rican, Cuban, South or Central American or other Spanish culture or origin?**
   ☐ Yes
   ☐ No

5. **During the pandemic, did you work or go to school? (Please select all that apply)**
   ☐ Working for pay
   ☐ On leave
   ☐ Laid off or lost job
   ☐ Unemployed and looking for a job
   ☐ Retired
   ☐ Staying at home / homemaker
   ☐ Disabled
   ☐ Enrolled in school/college/university

6. **Which best describes the area in which you live?**
   ☐ Large city
   ☐ Suburbs of a large city
   ☐ Small city
   ☐ Town or village
   ☐ Rural area

7. **What is the highest level of education you completed?**
   ☐ Some grade school

☐ Some high school
☐ High school diploma or GED
☐ Some college or 2-year degree
☐ 4-year college graduate
☐ Some school beyond college
☐ Graduate or professional degree

8. **What is your average household income per year in dollars?**
☐ Under $25,000
☐ $25,000 – $49,999
☐ $50,000 - $99,999
☐ $100,000 and over

9. **When did you start peritoneal dialysis?** _______ month _______ year
(e.g. December 2012)

10. **Are any adults living in the home an ESSENTIAL WORKER (e.g., healthcare, delivery worker, store worker, security, building maintenance)? Y/N**
☐ Yes
☐ No

If you answered 'Yes' to Question #10:
Did they come home most days of the week?
☐ Yes
☐ No

11. **How long does it take for you to get to the dialysis unit each time you go?**
_________hours_________mins

12. **Do you live by yourself?**
☐ Yes
☐ No

13. **Do you have a caregiver, friend, family member, or partner who helps you take care of anything related to your health?**
☐ Yes
☐ No

Between March 2020 and March 2021:

1) **In order to do your dialysis at home, did you have to leave your home or arrange for alternative accommodations?**

☐ Yes

☐ No

2) **If you had a job, did you work from home or did you have to go on-site? Please select all that apply**

☐ Work from home

☐ Work on-site

☐ A combination of working from home and on-site

☐ Other: ________________

☐ N/A

3) **Did you do your monthly visits using telehealth (Zoom, FaceTime, WhatsApp, telephone or other applications)?**

☐ Not at all

☐ Rarely

☐ Often

☐ All the time

4) **Did you have issues getting dialysis supplies delivered <u>TO</u> your home? (i.e. issues with deliveries not happening, or problems contacting the delivery driver)**

☐ Not at all

☐ Rarely

☐ Often

☐ All the time

5) **a) Did you have issues getting dialysis supplies carried <u>INSIDE</u> your home? (i.e. issues with getting the delivered supplies brought into your home)**

☐ Not at all

☐ Rarely

☐ Often

☐ All the time

b) Who brought your supplies into your home?

☐ Myself

☐ Family member

☐ Friend

☐ Delivery Driver

☐ Other

6) **How many times did you have trouble getting your medications?**

☐ Zero

☐ 1-4 times
☐ 5-7 times
☐ More than 8 times

7) Were you hesitant to come to the dialysis unit for your monthly visits?
☐ Not at all
☐ Rarely
☐ Often
☐ Always

8) Did you have times when you wanted to come to the unit for a dialysis-related issue, but didn't because of fear of COVID?
☐ Yes
☐ No

9) Did you feel supported by the home dialysis unit staff?
☐ Not at all
☐ Rarely
☐ Often
☐ All the time

10) Did you think about switching to in-center dialysis?
☐ Not at all
☐ Rarely
☐ Often
☐ All the time

11) If you were offered a transplant, would you have agreed to it?
☐ Yes
☐ No

12) To the best of your recollection, how many dialysis treatments did you miss?
☐ None
☐ 1-2 treatments/month
☐ 3-4 treatments/month
☐ > 4 treatments/ month

13) Did you have any issues getting a ride to the dialysis unit?
☐ Yes
☐ No

Between March 2020 and March 2021, how often have you been bothered by any of the following problems?

a) Little interest or pleasure in doing things

- ☐ Not at all
- ☐ Several days
- ☐ More than half the days
- ☐ Nearly every day

b) Feeling down, depressed, or hopeless

- ☐ Not at all
- ☐ Several days
- ☐ More than half the days
- ☐ Nearly every day

How often have you been bothered by the following problems?

a) Feeling nervous, anxious, or on edge

- ☐ Not at all
- ☐ Several days
- ☐ More than half the days
- ☐ Nearly every day

b) Not being able to stop or control worrying

- ☐ Not at all
- ☐ Several days
- ☐ More than half the days
- ☐ Nearly every day
